# The Uses of Freedom in Nursing Administration

**Published:** 1920-07-17

**Authors:** 


### THE MATRONS' AND SISTERS' DEPARTMENT.

### THE USES OF FREEDOM IN NURSING ADMINISTRATION.

British nursing has followed so unfettered a path in its various stages of development that one cannot see the approach of a centralised system without a kind of misgiving. It is true that there are many things which could be better ordered if control were unified. There is need for standardisation of training, for a general pension system, for a levelling up of salaries, for inspection of domestic conditions, and so on. But a comparison of the privileges enjoyed by nurses in voluntary institutions with those conferred under official control forbids the supposition that it is liberty which has produced abuses. It is rather the case that nursing started at a time when the standard of wholesome living for everybody was very far below what it is now, and that the reaction to better ways has been less impeded in voluntary institutions, unfettered by officialism, than in places where everything is wound up like a clock to go automatically.

We are far from believing that the nation has reached the limit of the changes for the better dictated by science and common sense. For that reason we should deprecate any system which might be devised by the Ministry of Health for placing nurses under a centralised authority and thus crystallising their conditions of service even under what might seem at the moment a truly enlightened rule. The experimental character of nursing demands complete liberty within certain limits necessary, we now find, for the protection of the women who make up the service. To draw up a comprehensive scheme which should prescribe for the working out in every particular of conditions of training, conditions of living, of payment, and retirement, might give us for a time a vast improvement. But almost before it got into working order the perfect scheme would crack somewhere. Contingencies which had never been foreseen would make some part of it unworkable and abuses would creep in. Or some very important new advance would be held up because the scheme had left no room for initiative.

Very few of the gradual improvements which have helped to make British nursing what it is would have taken place if it had been necessary to institute them as yet all untried simultaneously over the whole kingdom. None, if our memory serve us, were first initiated under the Poor-Law Service. They have been inspired by nurses working their way through manifold impediments to a position of authority in which they became free under enlightened lay control to abolish hardships in their nurses' training under which they themselves had once suffered, and try experiments in making nursing more efficient. It is true that progress has been slower this way than if by the stroke of a pen the reform could have been instituted in a thousand institutions. But the point is that progress, however slow, has been attained. Gigantic alterations which affect thousands have a way of never materialising. They lie hidden in piles of official memoranda and their fate is to assist in paving the lower regions.

A forcible illustration of the advantages of freedom is afforded in the simple instance of the catering arrangements under officialism and under voluntary management. The official theory is that each member of the staff is entitled to a specified quantity of specified articles of food and that none can be trusted to interfere in any way with their getting it. The quantity of each article is reckoned out and cooked, just this and nothing else, and all that the individual is permitted to do is to eat it. Ordinary managers merely throw on the matron or steward the responsibility for seeing that the staff are properly catered for, and they leave the person whom they place in authority full liberty to order what is necessary. The contrast in quality and price is instructive. The cost per head for catering in Poor-Law establishments used in pre-war days to be pretty exactly double that in voluntary institutions. In the one case the monotony of the diet, the absence of seasonal changes, and the poor cooking were notorious and certainly injured the service. In the latter, as at Guy's for instance, at less than half the cost the catering was on the level of a good hotel.

How could sister-tutors have come into being under a centralised authority? Impossible to decree "Let there be sister-tutors." The process of inaugurating the movement must begin in faith and proceed in patience. Impracticable also to introduce outside examiners all at once. This innovation had to be tried first, the schools prepared for it, and its effects observed. So with the fourth year of training, so with countless improvements in teaching and in the wards. We all want to get on more quickly and to stimulate the laggards, but is it always recognised that after all initiative is the mainspring of progress? Let us guard against its destruction.

